# Acknowledgement to Reviewers of *Microorganisms* in 2018

**DOI:** 10.3390/microorganisms7010013

**Published:** 2019-01-09

**Authors:** Microorganisms Editorial Office

**Affiliations:** MDPI, St. Alban-Anlage 66, 4052 Basel, Switzerland

Rigorous peer-review is the corner-stone of high-quality academic publishing. The editorial team greatly appreciates the reviewers who contributed their knowledge and expertise to the journal’s editorial process over the past 12 months. In 2018, a total of 127 papers were published in the journal, with a median time to first decision of 17 days and a median time to publication of 41 days. The editors would like to express their sincere gratitude to the following reviewers for their cooperation and dedication in 2018:
Abraham, Wolf-RainerLanza, GiuseppeAbu-Niaaj, LubnaLeekitcharoenphon, PimlapasAdhya, SankaLemos, Manuel L.Aitken, Michael D.Leuko, StefanAlduina, RosaLi, HongchunAlessi, AnnaLi, YvonneAl-Fatimi, MohamedLima, NelsonAlterskjær Sundset, MonicaLingwood, CliffordAnand, DeepakLucchesi, PaulaAncona-Contreras, VeronicaLuedtke, BrandonAndersen-Ranberg, Emilie U.Łukaszewicz, MarcinAndrás, AbonyiLuna, Gian MarcoAndreoni, FrancescaLuthra, AmitAntoninka, AnitaLyu, ZheAntunes, AndréMacrae, Jean D.Arthur, Terrance M.Madrid, CristinaArunkumar, AbhiramMakarewicz, OliwiaAsensio, MiguelMaki, JamesAtashgahi, SiavashMalins, LaraAuchtung, ThomasMancianti, FrancescaAzman, SametMancini, InesBader, OliverMandyam, KeerthiBae, TaeokMarcos, Jose F.Bagnoud, AlexandreMariño, ElianaBaldassarre, Maria ElisabettaMariottini, Gian LuigiBaleja, JamesMarnocha, CassandraBansal, NamitaMartinson, Guntars O.Bansil, RamaMcArthur, SimonBarba, Francisco J.McDevitt, Sylvia FrankeBarja, Juan L.McDowell, AndrewBaxter, BonnieMcEwan, NeilBeblo-Vranesevic, KristinaMcHardy, IanBecerra, ManuelMei, ChuanshengBej, AsimMellbye, Brett L.Benítez-Páez, AlfonsoMellies, JayBergen, MartinMemarzadeh, KavehBernal, PatriciaMenendez, EstherBerrue, FabriceMenon, VipinBersch, BeateMercier-Bonin, MurielBevilacqua, AntonioMercier-Bonin, MurielBiedendieck, RebekkaMetanis, NormanBomberg, MalinMeziti, AlexandraBosi, PaoloMirza, Babur S.Bosilevac, JosephMoll, Gert N.Bosma, Elleke FMorgan, RoderickBrautaset, TrygveMoschou, Panagiotis N.Brazelton, WilliamMoxley, RodneyBriandet, RomainMuschiol, JanBrumm, Phillip J.Mußmann, MarcBultman, TomMutharia, Lucy M.Callaway, Todd RileyNakai, RyosukeCampana, RaffaellaNakajima, MasamiCaneiras, CátiaNaughton, LynnCardona, LluisNett, MarkusCarella, PhilipNett, MarkusCarson, Christine F.Neubauer, PeterCaruso, GabriellaNguyen, PeterCassard-Doulcier, Anne MarieNguyen, Van KhanhCastanera, RaúlNicaud, Jean-MarcCataldi, AngelNicoletti, Vincenzo G.Cavalca, LuciaNielsen, Peter E.Cenci-Goga, Beniamino T.Nilsson, HenrikCerenius, LageNishiyama, KeitaCeriotti, AldoNiu, DongyanChandler, MichaelNoack, StephanCharpentier, XavierOgunade, IbukunChen, HaoyuanOsorio, CarlosChistoserdova, LudmilaOuwerkerk, DianeChronopoulou, MyrsiniOvery, David P.Cizeikiene, DaliaPace, FabioCollins, JamesPalmer, JonCompant, t StephanePalmer, Robert J.Conti, StefaniaPanico, AntonioCooney, Charles L.Papamichael, EmmanuelCorfield, Anthony P.Park, Hyun-WooCorfield, TonyParrilli, ErmenegildaCovasa, MihaiPascual, JavierCox, Jonathan A.G.Patarata, LuísCuesta, IsabelPaterson, RussellCurtis, WaynePatil, SonalDa Costa, MiltonPhalen, DavidDaly, KristianPieper, RobertDalziel, Julie EPopp, DennyDame, Remus T.Porro, DaniloD'Argenio, ValeriaPorta, AmaliaDavid, Michael Z.Postec, AnneDe Berardinis, PiergiuseppePotter, BethDearing, DenisePurkamo, LottaDegenkolb, ThomasPuzon, Geoffrey J.Desmet, TomRaap, JanDimitrellou, DimitraRadcliff, Fiona JaneDionisio, GiuseppeRahman, Khondokar MizanurDishaw, LarryRai, NavneetDong, XiyangRajala, PauliinaDörr, MarkRamirez-Paredes, Jose GustavoDramsi, ShaynoorRatkowsky, David A.Drosinos, Eleftherios H.Ravi, AnuradhaDunfield, PeterReed, David W.Economou, VangelisReed, Kevin F. M.Edwards, MartinRehman, Junaid U.El Aidy, SaharReid, Christopher W.Ellegaard, MarianneRendueles, ManuelElleuche, SkanderRichard, PeterEl-Matbouli, MansourRizzo, Peter J.Enjalbert, FrancisRocha-Martin, JavierErb, TobiasRodrigo, Sara MoralesEyice-Broadbent, OzgeRogozhin, EugeneFalconi, MaurizioRuffino, BarbaraFardeau, Marie-LaureRuiz, NicolasFeil, Edward J.Russo, PasqualeFein, Jeremy B.Sabet, ShereenFernandez-Martinez, LorenaSadykov, Marat R.Ferreira, Rui M.Sagona, AntoniaFioretto, AntoniettaSambri, VittorioFischer, DoreenSanso, Andrea MarielFleshner, MonikaSantiago-Rodriguez, Tasha MarieForsyth, Christopher B.Sass, HenrikFrançois Pouchus, YvesSavino, FrancescoFreimoser, FlorianScala, ValeriaFujiwara, ShinsukeSchäberle, Till F.Futoma-Kołoch, BożenaSchimek, ChristineGabriel, KyleSchoepp-Cothenet, BarbaraGarbowska, MonikaSeetharam, ArunGeerlof, ArieSegura, AnaGomes, Maria S.Setzer, William N.Gómez, FernandoShcherbakova, ViktoriaGómez, MarisaSherchan, SamendraGonçalves, PaulaShoguchi, EiichiGonzález-Siso, María-IsabelShoham, MenachemGoossens, KarenShubitz, Lisa F.Gornik, SebastianSimões, NelsonGraziano, GiuseppeSingh, Om V.Greetham, DarrenSkaar, IdaGrellier, PhilippeSmedile, FrancescoGrosjean, HenriSmith, KirstyGrosse, StephanSmith, Sinead M.Gundogdu, OzanSmith, Thomas J.Hahn, MartinSpanbauer, TrishaHale, Vanessa L.Stadler, MarcHammer, KatherineStanford, KimHansford, KarlStenberg, ReidunHarada, KazukiStevens, ColeHardwidge, PhilipStingl, UliHarijan, Rajesh K.Stokke, RunarHendry, Tory A.Stromberg, ZacharyHenry, Kathene JohnsonTalon, RégineHerrero, EnriqueTan, ZhongpingHolmes, Dawn ETani, AkioHolt, Scott M.Tassou, ChrysoulaHomayouni, AzizTengs, TorsteinHötzinger, MatthiasThies, StephanHovde, CarolynThornton, DavidHrdý, JiříThullner, MartinHuang, I-HsiuTigini, ValeriaHuang, LinfengTiscar, Pietro G.Hurley, JamesTischler, DirkIatsenko, IgorTkavc, RokIebba, ValerioTohno, MasanoriInsam, HeribertTolker-Nielsen, TimIshaq, Suzanne LynnTorres, Adrian GabrielJay-Russell, MicheleTreuner-Lange, AnkeJehmlich, NicoTreusch, Alexander H.Jena, Prasant KumarTuovinen, OlliJenkins, ClaireTurnau, KatarzynaJorand, Frédéric P.A.Turroni, SilviaJoseph, SusanTurton, JaneJu, Kou-SanUrbanczyk, HenrykKalatzis, Panos G.Vallini, GiovanniKallas, ToivoVan Kranenburg, RichardKalsi, MeghaVavitsas, KonstantinosKamo, KathrynVega Gutierrez, SarathKanamori, TakaoVelin, DominiqueKarayanni, HeraVillaverde, AntonioKatsifas, Efstathios A.Villena, JulioKatyal, PriyaViñas, MiguelKaushik, AshleshaWaldbieser, Geoffrey CKelly, VincentWaller, RossKim, ChyerWang, YimingKim, HoonWang, YuqiKim, Kyung MoWasmund, KennethKimura, Ken-ichiWemheuer, BerndKleiner, ManuelWheelhouse, NickKlemenčič, MarinaWhiteson, KatrineKneil, KaliWhitworth, David E.Knott, EmilyWielgoss, SébastienKöck, RobinWilharm, GottfriedKocsubé, SándorWojtyczka, RobertKöller, ManfredWong, AdamKomaki, HisayukiWood, James B.Koulenti, DespoinaWöstemeyer, JohannesKovbasnjuk, OlgaWu, NaichengKowalczyk, PawełWu, ZuoweiKracher, DanielYamamoto, KenKuenz, AnjaYan, XiaLajhar, Salma A.Yang, WeishanLalor, Stephen J.Yilmaz, BahtiyarLaMontagne, Michael G.Zhang, RuiyongLang, ElkeZhu, JingenLangley, SeanZiarno, Małgorzata

